# Effects of Harvesting Site and Incision Method on Surgical Wound Complications of No-Touch Saphenous Vein Grafts: A Retrospective Observational Study

**DOI:** 10.21470/1678-9741-2024-0098

**Published:** 2025-05-23

**Authors:** Hironobu Sakurai, Dai Tasaki, Tomoya Yoshizaki

**Affiliations:** 1 Department of Cardiovascular Surgery, Musashino Red Cross Hospital, Tokyo, Japan

**Keywords:** Coronary Artery Bypass, Saphenous Vein, Wounds and Injuries, Coronary Vessels

## Abstract

**Introduction:**

Saphenous vein grafts are frequently used for coronary artery revascularization. However, harvesting veins is associated with infected surgical sites and other complications. The no-touch technique that includes harvesting saphenous vein grafts along with surrounding tissues improves graft patency but increases the frequency of wound complications. We harvested saphenous vein grafts using the no-touch technique and devised other options for sites and incision methods to prevent wound complications. This study aimed to determine the clinical outcomes of no-touch saphenous vein grafts as well as associations between harvesting methods and wound complications.

**Methods:**

We enrolled 132 patients who underwent isolated coronary artery bypass surgery with saphenous vein grafts harvested using the no-touch technique. Wound condition, general status, and graft patency were assessed during clinical follow-up.

**Results:**

We harvested 180 veins (lower legs, n = 69 veins; upper legs, n = 111) using longitudinal and skip incisions at 100 and 80 sites, respectively. Wound complications occurred at 35 sites. The frequency of complications was significantly lower in the upper, than in the lower legs (14.4% vs. 27.5%). Furthermore, wound complications were reduced more by skip, than by longitudinal skin incisions (16.3% vs. 20.0%).

**Conclusion:**

We devised a method to harvest no-touch saphenous vein grafts and determined the clinical outcomes of saphenous vein grafts and harvesting sites. Harvesting from the upper leg and via skip incisions reduced the frequency of wound complications.

## INTRODUCTION

**Table:** 

Abbreviations, Acronyms & Symbols	
BITA	= Bilateral internal thoracic artery
BMI	= Body mass index
CABG	= Coronary artery bypass grafting
EVH	= Endoscopic vessel harvesting
IABP	= Intra-aortic balloon pump
LAD	= Left anterior descending artery
NPWT	= Negative pressure wound therapy
NT	= No-touch
SD	= Standard deviation
SVGs	= Saphenous vein grafts

**Vídeo 1:**
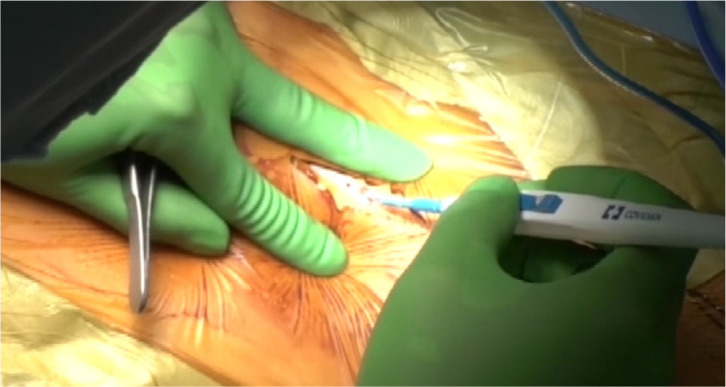
Harvesting the saphenous vein from the upper leg with skip skin incision. Link: *https://youtu.be/Lv22wNYpiMU*

Saphenous veins run from the lower legs to the thighs and are commonly used as bypass conduits for coronary artery bypass grafting (CABG) as they are accessible and long enough for coronary artery revascularization. However, the patency of saphenous vein grafts (SVGs) is not as good as that of other conduits, and wound complications often occur at harvesting sites^[1^-^3]^. Efforts to improve SVG patency include graft design, graft assessment, and postoperative medical therapy^[4^-^6]^. Harvesting SVGs can also be associated with complications such as infection, lymphorrhea, and dehiscence. Severe complications require additional treatment but rarely lead to limb loss. The no-touch (NT) technique is a novel method for harvesting SVGs together with their surrounding tissues; however, it exacerbates wound complications.

The patency of NT SVGs is superior to that of the radial arteries and might be comparable to that of the internal thoracic artery^[4^,^7^,^8]^. Surrounding tissues might contribute to preserving the vessel wall structure and vasa vasorum and prevent vein kinking. The good patency and clinical benefits of the NT technique were reported in the 2018 European Society of Cardiology and European Association for Cardiothoracic Surgery guidelines on myocardial revascularization (class of recommendation IIa, level of evidence B)^[9]^. However, the NT technique is associated with more frequent wound complications than the conventional technique owing to a deficiency in surrounding tissues^[10^-^12]^. Several studies have introduced modified methods to address this issue^[4^,^13^,^14]^.

We harvested SVGs using the NT technique and devised a method to prevent wound complications. This study aimed to determine the clinical outcomes of NT SVGs as well as associations between harvesting methods and wound complications.

## METHODS

### Ethical Statement

This study was approved by the Institutional Review Board of Musashino Red Cross Hospital (approval no: 5011; April 24, 2023), and all patients provided written informed consent to participate.

### Coronary Artery Bypass Grafting Using No-Touch Saphenous Vein Grafts

Patients were usually treated by off-pump CABG, or with an intra-aortic balloon pump for cardiopulmonary bypasses in beating or arrested hearts when hemodynamics were unstable. The right coronary artery, left circumflex artery, or diagonal branch was revascularized using SVGs. Grafts were anastomosed proximally to the aorta and distally to target coronary arteries. Several stenotic sites were sequentially anastomosed. We excluded patients with contraindications for SVG harvesting, such as those with an ankle-brachial index < 0.5, veins with varices and abnormal courses, or narrow veins with a short diameter.

The NT technique proceeded as follows (Supplementary Video):

The saphenous veins are preoperatively assessed using contrast-enhanced or computed tomography to assess the course and diameter of the veins and identify varicose veins or large side branches.

**Fig. 1:**
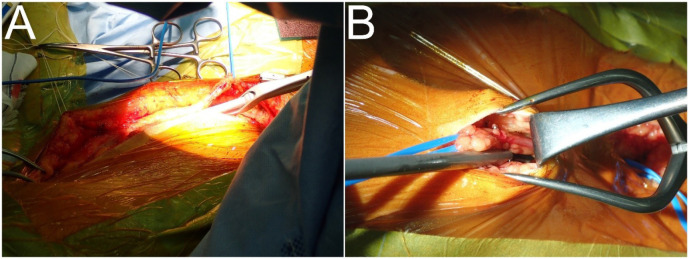
Ultrasonic scalpel for harvesting saphenous vein from the upper leg using the no-touch technique.

Continuous longitudinal or skip incisions are made in the skin (Figures 1A and B, respectively). The anterior surface of a vein is exposed throughout the entire length of the lower or upper legs leaving connective tissues intact.

After skip incisions, a vein under a skin bridge is harvested along 5-mm margins of adipose tissue on both sides using a harmonic scalpel ACE+ with a 23 cm shaft (Ethicon Endosurgery, Inc., Cincinnati, Ohio, United States of America) (Figure 1A)^[15]^. The posterior surface of the vein is then stripped, leaving a connective tissue.

An active clotting time of 200 seconds was maintained with systemic heparin sodium (4000 IU). The vein was marked with ink to prevent twisting. The harvested vein was connected to a 5-Fr sheath inserted into the femoral artery for spontaneous dilation under arterial pressure. Bleeding from the lateral branches was simultaneously assessed. The vein was stored in moist milrinone-soaked gauze until anastomosis.

### Leg Wound Management

After achieving hemostasis at skin incision sites, wounds were irrigated using isotonic sodium chloride and then closed in two layers using 3-0 absorbable monofilament running sutures. The skin was finally closed using a skin stapler. Postprocedural bleeding and fluid collection were prevented using an elastic bandage^[4^,^14]^.

We assessed wound healing and complications. All patients were followed up at one month after discharge, at six and 12 months postoperatively, and annually thereafter.

A leg wound complication was defined as requiring additional treatment^[16^,^17]^. Suspected infections were treated with intravenous or oral antimicrobial agents, which were terminated when infections and inflammation were resolved. Infected or necrotic tissues were debrided. Small tissue defects caused by surgical debridement and wound dehiscence were irrigated with 0.9% sodium chloride and treated with ointment. The surgical wounds were closed according to their condition. Large tissue defects were irrigated with isotonic sodium chloride, and negative pressure wound therapy (NPWT) was applied. Lymphorrhea was treated by compression or wound closure.

### Clinical Outcomes

All enrolled patients were clinically followed up by coronary angiography or multi-slice coronary computed tomographic angiography before discharge, one year postoperatively, and annually thereafter to assess leg wound healing and functional outcomes. Graft patency was graded according to the FitzGibbon classification^[18]^ as excellent with unimpaired runoff (A), patent but with < 50% stenosis (B), or occluded (C). Competitive graft flow was classified as grade B. Grades A and B were considered patent, whereas grade C was considered occluded. Patients with renal dysfunction, allergies to contrast dyes, or who refused to undergo assessment using these modalities were assessed by myocardial scintigraphy.

We assessed the frequency of wound complications at harvest sites in the upper and lower leg, and differences between longitudinal and skip incisions. The patients were assigned to early or late groups based on the timing of surgery, and differences between them were analyzed.

### Statistical Analyses

All data were statistically analyzed using R version 3.6.1 (R Foundation for Statistical Computing, Vienna, Austria). Clinical outcomes were statistically analyzed using Fisher’s exact test for 2 × 2 tables. Values with *P* < 0.05 were considered statistically significant.

## RESULTS

### Clinical Outcomes

Among the 215 patients who were treated by isolated CABG at our institution between October 2017 and January 2023, 191 underwent isolated CABG using SVGs, and 180 veins were harvested from 132 of them using the NT technique. The median follow-up time was 20.9 (interquartile range, 11.8-36.1) months. Postoperative graft patency rates were assessed in 120 and 77 patients before discharge (early) and one year later, respectively.

Table 1 shows perioperative data of the patients. One patient died perioperatively due to an acute subdural hematoma. Perioperative complications that occurred in 18 patients comprised acute renal failure requiring new dialysis (n = 2), Coronavirus disease 2019 (n = 1), cerebral hemorrhage (n = 2), mediastinitis (n = 3), hemorrhage requiring reopening the chest (n = 2), pneumothorax (n = 4), reintubation (n = 3), and necrotizing cholecystitis (n = 1). Leg wound complications developed in 29 patients.

**Table 1:** Demographic information and perioperative data of patients who underwent coronary artery bypass grafting with saphenous vein grafts harvested using the no-touch technique.

	Patients (n = 132)
Preoperative characteristics	
Age (years), mean ± SD	67.8 ± 9.8
Older individuals (> 74 years), n (%)	36 (27.3%)
Female, n (%)	21 (15.9%)
Obesity (BMI > 25 kg/m^2^), n (%)	41 (31.1%)
Hypertension, n (%)	107 (81.1%)
Dyslipidemia, n (%)	108 (81.8%)
Diabetes mellitus, n (%)	62 (47.0%)
Insulin-dependent, n (%)	27 (20.5%)
Chronic kidney disease, n (%)	54 (40.9%)
Hemodialysis, n (%)	4 (3.0%)
Corticosteroid administration	1 (0.8%)
Operative information	
Off-pump CABG	131 (99.2%)
IABP	77 (58.3%)
Number of anastomoses (mean ± SD)	4.1 ± 1.0
BITA	10 (7.6%)
Postoperative data	
Hospital death	1 (0.8%)
Death during follow-up	6 (4.5%)
Complications	48 (36.4%)
Leg wound complications	29 (22.0%)

BITA=bilateral internal thoracic artery; BMI=body mass index; CABG=coronary artery bypass grafting; IABP=intra-aortic balloon pump; SD=standard deviation

Table 2 shows information about the 180 SVGs harvested using the NT technique from the lower (n = 69) and upper legs (n = 111) for 138 and 204 anastomoses, respectively. Continuous longitudinal and skip incisions were made at 100 and 80 sites respectively, and subcutaneous drains were inserted at 147 of them. Among 341 anastomoses with 180 SVGs (mean, 1.9 anastomoses), 67 and 123 veins were used for sequential and single anastomoses, respectively. Six SVGs were connected to artery conduits as composite grafts. Table 2 shows the SVG target arteries and graft patency rates.

**Table 2:** Data of saphenous vein grafts harvested using the no-touch technique.

	Grafts (n = 180)
Harvesting site	
Lower leg	69
Upper leg	111
Incision type	
Longitudinal incisions	100
Skip skin incisions	80
Number of anastomoses, mean ± SD	1.9 ± 0.7
Target vessel	
Right coronary artery	78
Left circumflex artery	192
Diagonal branch	71
Left descending artery	
Graft patency (FitzGibbon grades A and B)	
Early (before discharge)	96.5% (302/313)
One year postoperatively	88.9% (176/198)

SD=standard deviation

### Wound Complications

**Fig. 2:**
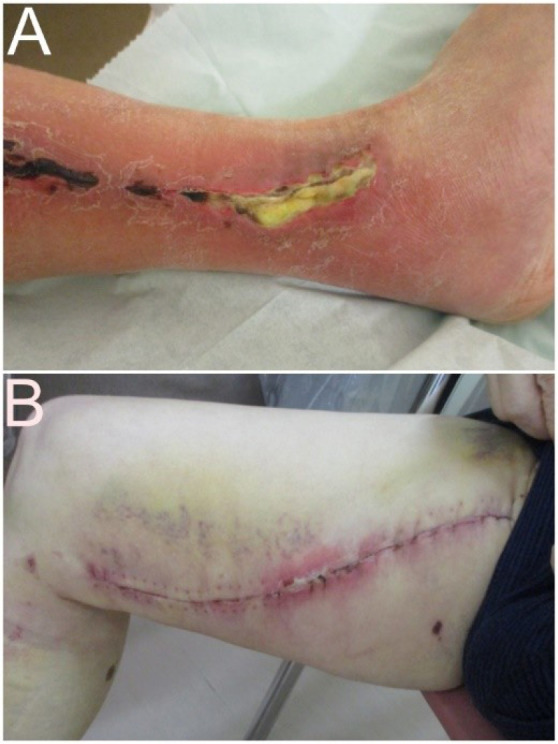
Wound complications (A) Infection at lower leg treated with debridement and negative pressure wound therapy. (B) Necrosis at site in upper leg treated with ointment.

**Fig. 3:**
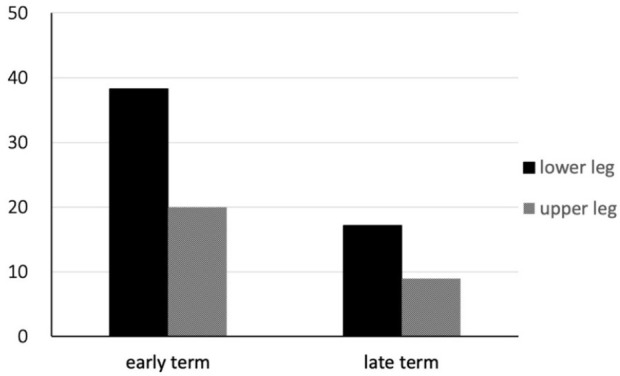
Frequency of wound complications in lower and upper legs differ between assessments before (early) and after (late) discharge.

Table 3 shows that wound complications requiring additional treatment at 35 (19.4%) harvested sites in 29 patients comprised infection (n = 18; Figure 2A), necrosis (n = 9; Figure 2B), lymphorrhea (including three with comorbid infection) (n = 7), and dehiscence (n = 4). Further treatment included NPWT (n = 13), debridement (n = 6), antimicrobial agents (n = 9), and others (n = 7). All complications were resolved during follow-up. Wound complications occurred more frequently at sites in the upper, than in the lower legs (16 [14.4%] *vs.* 19 [27.5%]; *P* = 0.03). The frequency of complications did not significantly differ between longitudinal and skip incisions at 22 (20.0%) and 13 (16.3%) sites, respectively. The frequencies of wound complications in the early and late groups were respectively 13 (38.2%) of 34 *vs.* six (17.1%) of 35 (*P* = 0.06) sites in the lower legs and 11 (20.0%) of 55 *vs.* five (8.9%) 56 sites (*P* = 0.11) in the upper legs, respectively (Figure 3). Although the frequency of complications did not significantly differ between the lower and upper legs, it decreased at both sites.

**Table 3:** Leg wound complications of saphenous vein graft harvesting sites.

	Lower leg (n = 69)	Upper leg (n = 111)	*P*-value
All complications	19 (27.5%)	16 (14.4%)	0.03
Infection	11 (15.9%)	7 (6.3%)	
Necrosis	3 (4.3%)	6 (5.4%)	
Lymphorrhea	4 (5.8%)	3a (2.7%)	
Dehiscence	1 (1.4%)	3 (2.7%)	
Therapy			
Negative pressure wound therapy	7	6	
Debridement alone	3	3	
Antimicrobial administration alone	5	4	
Others	1	3	

a Overlap with infection

**Fig. 4:**
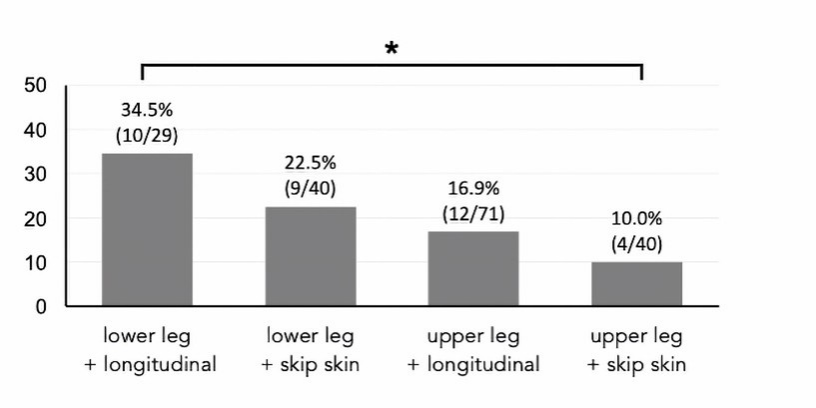
Differences in frequency of wound complications between lower and upper legs and longitudinal and skip incisions. Longitudinal incision of lower leg and skip incision of upper leg significantly differ. *P = 0.017.

Figure 4 shows the frequency of wound complications according to differences in harvesting sites (lower or upper leg) and longitudinal or skip incisions. The frequency of wound complications among the four groups was the lowest in the upper legs with skip incisions (10.0%) and significantly lower than in the lower legs and with longitudinal incisions (34.5%).

## DISCUSSION

We harvested SVGs using the NT technique with a harmonic scalpel and devised techniques for harvesting and to reduce wound complications. The patency of SVGs was similar to that of previous early, mid-term (1-5 years), and long-term (> 5 years) graft patency rates of 94.3%-100.0%^[4^,^13]^, 90.6%-95.4%^[7^,^10^,^12]^, and 83.0%-91.0%^[7^,^8]^, respectively. Moreover, the present study revealed a significantly lower frequency of wound complications in the upper than in the lower legs.

The internal thoracic artery is the gold standard conduit for revascularizing the left anterior descending artery (LAD) during CABG and it has significantly improved prognoses. However, a second-line conduit for non-LAD targets has been debated. Readily available, easily harvested SVGs are commonly used for CABG because they are long enough for grafting. Although the patency of SVGs has not reached that of arterial conduits, it has improved^[4^-^6]^. The NT technique was developed to harvest SVGs along with the perivascular tissues. Few studies have found that the patency of NT SVGs exceeds that of conventional SVGs and is comparable to that of arterial conduits. However, leg wound complications due to perivascular tissue deficiencies are a matter of concern. Therefore, the NT technique has been modified to address this issue^[4^,^13^,^14]^. Min-Seok Kim et al.^[14]^ described comprehensive strategies for wound complications and highlighted the importance of preoperative evaluation of saphenous veins using computed tomography or echography; moreover, they described appropriate methods for skin incision and vein harvesting as well as wound closure and postoperative treatment.

A tissue deficiency at harvesting site delays wound healing. Lower leg incisions are at risk of wound breakdown due to poor tissue quality, particularly around the medial malleolus^[16]^. We believe that tissue loss significantly affects the lower leg, which has less adipose tissue than the upper leg. Moreover, SVGs harvested from the upper leg have advantages for revascularization. These SVGs are appropriately thick and long enough to revascularize any coronary artery in most patients, including women, whereas those from the lower leg are thin and occasionally unsuitable for grafting.

Advanced age, female sex, diabetes mellitus, malnutrition, and chronic steroid therapy are risk factors for infections of harvesting sites^[16^,^19]^. The incision length is also associated with risk of wound complications. A systematic review of SVG harvesting for lower-extremity arterial bypass found fewer infections at harvesting sites with skip than with continuous incisions^[20]^. The frequency of wound complications is lower when the NT technique is applied with skip (2.9%-5.7%)^[4^,^13]^ than with continuous (11.1%-23.3%)^[10^-^12]^ incisions.

Our method differs from the original in that we use a harmonic scalpel to dissect the surrounding tissue. This allows easier and more effective fat tissue dissection, and it seals venous side-branches and lymph vessels. Furthermore, it reduces thermal injury to veins compared with electrocautery. Other methods have applied NT SVG harvesting using the ultrasonic scalpel of a bipolar device^[13]^. The harmonic scalpel series is used to harvest saphenous veins at our institution. These devices are widely used in endoscopic surgery and for harvesting arterial conduits^[2]^.

The harmonic scalpel has high burst pressure, enabling effective sealing, and less lateral thermal spread, which should reduce thermal injury to surrounding tissues^[15]^. Veins were harvested with a margin of ~ 5 mm on both sides to ensure sufficient distance and thus influence from the thermal source^[15]^. The effects of thermal injury on NT SVGs harvested using an ultrasonic scalpel are histologically negligible^[21]^, as the wall architecture, particularly that of the medial smooth muscle cells, is normal, and perivascular tissues are preserved. These morphological features are similar to those of SVGs harvested using the original NT technique^[11^,^13^,^22]^.

We assumed that the NT technique using an ultrasonic scalpel would be in line with the original NT technique. The device potentially helps to decrease wound complications and eases NT technique application. A scalpel with a long shaft can facilitate vein harvesting under subcutaneous tunnel sites after skip incisions. The frequency of wound complications was similar between the present and other findings, but it decreased over time. The NT technique has a gradual learning curve indicating that technical maturity will probably help to reduce wound complications.

The use of an ultrasonic scalpel device is appropriate for endoscopic vessel harvesting (EVH) and should reduce wound complications^[20^,^23^,^24]^. However, EVH is also associated with decreased graft patency^[23]^, which is likely due to difficulties with acquiring the endoscopic skills needed for harvesting vein grafts. The guidelines recommend that experienced surgeons should conduct EVH^[9]^. The patency rate is similar between skip and continuous incision harvesting, and the low rates of wound complications are comparable to those of EVH^[20]^.

Leg wound complications require additional interventions, including debridement, NPWT, and amputations^[16^,^17]^ when conservative treatments are inadequate. None of our patients required amputation, and all complications were resolved during follow-up. Patients with extensive tissue loss due to debridement received NPWT. Currently, NPWT plays an important role in wound therapy and it is applied during cardiovascular surgery to promote wound healing, to reduce wound complications and infections^[25]^, as well as to prevent leg wound complications.

### Limitations

This retrospective, observational study conducted at a single institution had several limitations. The study design conferred potential risk of bias in evaluating clinical outcomes. Clear criteria for choosing longitudinal or skip incisions have not been established. Comparing graft patency between upper and lower legs was hampered by the lack of information regarding SVGs needed for each coronary artery when SVGs were obtained from both sites. Moreover, the number of graft patency evaluations at one year postoperatively was low due to insufficient follow-up, chronic kidney disorders, and contrast dye allergies. We did not compare SVGs harvested using conventional techniques due to possible sources of error from different surgical durations.

## CONCLUSION

Surgical site infections and other complications are associated with harvesting saphenous veins for revascularization. The NT technique improves SVG patency; however, it increases the risk of wound complications. We devised a method to harvest NT SVGs and assessed the clinical outcomes of SVGs and harvesting sites. We found that the frequency of wound complications was lower when SVGs were harvested from the upper leg using skip incisions than from the lower leg using continuous incisions. In future, EVH for NT SVGs and NPWT on closed wounds can potentially reduce the frequency of wound complications.
